# Ibuprofen-Loaded, Nanocellulose-Based Buccal Films: The Development and Evaluation of Promising Drug Delivery Systems for Special Populations

**DOI:** 10.3390/gels11030163

**Published:** 2025-02-24

**Authors:** Katarina Bolko Seljak, Blaž Grilc, Mirjana Gašperlin, Mirjam Gosenca Matjaž

**Affiliations:** Faculty of Pharmacy, University of Ljubljana, Aškerčeva cesta 7, 1000 Ljubljana, Slovenia; katarina.bolko-seljak@ffa.uni-lj.si (K.B.S.); blaz.grilc@ffa.uni-lj.si (B.G.); mirjana.gasperlin@ffa.uni-lj.si (M.G.)

**Keywords:** buccal films, hydrogels, mucoadhesion, nanocrystalline cellulose, SMEDDS, ibuprofen, drug delivery, microemulsion

## Abstract

The objective of this work was to investigate the use of nanocrystalline cellulose (NCC) as a drug-delivery excipient for buccal films. Gel-like dispersions were created by blending either gel or powder NCC (gNCC or pNCC) with natural polymers (alginate, pectin, or chitosan) in water, with glycerol serving as a plasticiser. Ibuprofen (IBU) as an active pharmaceutical ingredient (API) was dissolved in a self-microemulsifying drug delivery system (SMEDDS) to improve its solubility prior to its addition to gel-like dispersions. Dispersions were dried, and resulting films were cut to 3 cm × 1.5 cm size, appropriate for buccal delivery. Rheological measurements revealed that shorter, thinner, and less crystalline nanocellulose fibres are more favourable for stronger gel properties. While overall, weaker gel structure prior to film casting also resulted in shorter disintegration time, this was not the case for NCC–chitosan films; here, the low solubility of chitosan in neutral media proved to be the main obstacle. Nevertheless, the prolonged disintegration of NCC–chitosan films did not impact the dissolution of IBU, as these films exhibited the fastest dissolution rate, followed by NCC–pectin and NCC–alginate. Furthermore, NCC properties significantly influenced the dissolution behaviour of the chitosan formulations, with gNCC favouring faster IBU release due to weaker gel formation prior to film casting.

## 1. Introduction

Buccal administration is acknowledged as an alternative route for the systemic delivery of small molecules as well as biologics compared to conventional oral or intravenous route with various delivery systems investigated and/or commercially available, including (mucoadhesive) buccal tablets and buccal films, sprays, as well as dissolvable microneedles. Among these formulations, films have gained relevance as one of the latest innovations in the field of pharmaceutical forms, being patient-friendly and convenient, especially for children, the elderly, patients with Parkinson’s disease, and patients after anaesthesia [1,2]. Namely, safe swallowing is the key formulation factor in designing medicine for these patients. Buccal films as mucoadhesive oromucosal preparations are designed to specifically attach to the oral mucosa and controllably release the active pharmaceutical ingredient (API) [3]. This successfully addresses patient populations with swallowing deficiencies; moreover, their small size and minimal thickness allow for simple administration with no liquid needed, altogether contributing to improved patient compliance and adherence. Furthermore, the flexibility of buccal films in comparison to buccal tablets is purported to be the reason for them being preferred by children and the geriatric population, along with their smaller and customisable size [4,5,6,7]. While there is a gap in the literature comparing the in vivo performance of oromucosal films with the conventional oral dosage form of a tablet, ropinirole oral films have been shown to significantly improve their bioavailability after both sublingual and buccal administration [8].

When designing film-based formulations for oral administration, it is important to distinguish between orodispersible and buccal formulations [9]. Orodispersible films are designed to disperse immediately, with average disintegration time being below 60 s, usually around 30 s, resulting in rapid onset of action following subsequent absorption, primarily in the gastrointestinal tract. Buccal films, on the other hand, dissolve in the oral cavity to deliver active substances over an extended period of time either for oral local therapy or transmucosal administration by direct absorption through the venous system that drains from the cheek, avoiding the first pass effect and enzymatic degradation [3,10,11]. With various methods employed for manufacturing, including solvent casting, hot melt extrusion, inject printing, and 3D printing, buccal films consist of single or, more often, multilayer sheets of suitable materials with hydrophilic polymers as the main constituent, which upon wetting with the saliva produce a hydrogel that adheres to the buccal mucosa. Typically, a mixture of polymers, rather than a single polymer, is utilised to produce films with suitable characteristics (e.g., mechanical strength, among others), a favourable drug release profile, and commonly mucoadhesive properties as well [1]. At this point, over thirty mucoadhesive polymers have been identified in film formulations, with various cellulose derivatives, polycarbophil, poly(ethylene oxide), poly(meth)acrylic acid, chitosan, polyvinyl alcohol, polyvinyl pyrrolidone, gelatin, sodium alginate, gellan gum, carrageenan, pectin, hyaluronic acid, various starches, and pullulan among the most often used, with included plasticisers in order to regulate mechanical properties, e.g., folding capacity, tensile strength, toughness, hardness, and elastic modulus (glycerol, propylene glycol and/or polyethylene glycols being recurrently used), as thoroughly reviewed in [6,12].

Nanocrystalline cellulose (NCC) is a natural polymer that does not exceed 100 nm in at least one dimension. This nanoform structure is obtained through a specific processing of cellulose, using several mechanical, chemical, and enzymatic or a combination of these methods. The immense potential of NCC is directly connected to the superior properties of cellulose as its starting compound, such as being recognised as a biocompatible and biodegradable material, plus the low cost of NCC production from the most abundant biopolymer in nature, which all together aligns with the current orientation towards renewability and sustainability paradigm [13]. Consequently, numerous potential biomedical applications of NCC have been exploited, i.e., for diagnosis and biosensing, vascular graft replacement, tissue engineering in general, as viral inhibitors and support for the immobilisation of enzymes/proteins, and as a drug delivery excipient for cancer target therapy, as thoroughly reviewed by Karimian et al. [14]. More specifically, within the field of pharmaceutical technology, NCC expands the set of polymers for either the binding and controlled release of drugs of [15] or as a main constituent for oral and (trans)dermal dosage forms, utilising new advances such as the high viability of a probiotic bacterium when incorporated in tablet formulation based on alginate, pectin, and cellulose nanocrystals [16], or improved outcomes in atopic skin for film-forming hydrogels with NCC in combination with alginate or/and pectin [17], while bio-nanocomposite films of chitosan and poly (vinyl pyrrolidone), when combined with nanocellulose, showed improved structural and efficacy characteristics as an eventual wound dressing material [18].

Ibuprofen (IBU) is a commonly used nonsteroidal anti-inflammatory drug to relieve pain, fever, and inflammation. To achieve systemic action, IBU is typically administrated orally; however, due to its pre-systemic metabolism in the liver, other administration routes have been sought, especially for specific effects/target sites such as its neuroprotective effect in brain tissue, where promising results were obtained following the intranasal administration of mucoadhesive microemulsion [19]. In addition, IBU as a Class 2 API with low solubility but high permeability necessitates approaches to increase solubility [20]. Regarding films as potential drug delivery systems for ibuprofen-controlled release, poly(lactic-co-glycolic acid)-based films were developed, where high loading of IBU was explained in relation to its solubility in the polymer, with the release rate being dependent on its loading content as well as the pH of the released mediums’ relationship with solubility [21]. Another potential improvement offered by the buccal delivery of ibuprofen is at least partially avoiding the GIT absorption of ibuprofen and, in turn, lessening the appearance of GIT complications, typically experienced by 15–30% of patients utilising non-steroidal anti-inflammatory drugs long term [22].

Within the proposed study, nanocellulose-based buccal films were designed for the incorporation of IBU in order to prepare feasible and ready-to-use formulation, being of special benefit, especially for paediatric and geriatric populations, in addition to an improved IBU safety profile by avoiding oral intake-related side effects. Buccal films were based on two NCCs from different manufacturers, supplied in either gel or powder forms (gNCC or pNCC). Additionally, one of the natural polymers (alginate, pectin, or chitosan) was added to the formulation. To achieve suitable drug dosage, the IBU was previously dissolved in a self-microemulsifying drug delivery system (SMEDDS) that was subsequently incorporated into film-casting hydrogels. The assessment of hydrogels included differential scanning calorimetry (DSC) and an investigation of the NCC type on the rheological properties of film-casting hydrogels. Subsequently, the solvent evaporation method was utilised to prepare buccal films. Finally, NCC-based buccal films were evaluated for IBU in vitro release using an advanced flow cell relevant to the formulation as well as application site.

## 2. Results and Discussion

### 2.1. Development of Film Casting Formulations and IBU Incorporation

Film-based drug delivery systems address several current challenges in drug delivery. Unfortunately, due to their size limitations, such systems are often hampered by the low volume available for API loading [23]. This problem is further exacerbated when developing drug delivery systems for poorly soluble APIs, as films are commonly based on hydrophilic polymers. Moreover, crystallisation of the amorphous API in the polymer matrix of the films has been described, which could also further exacerbate dissolution problems of poorly soluble API [24]. Dissolving the API in SMEDDS prior to its incorporation in bioadhesive hydrophilic drug delivery systems enabled us to apply the advantages of these formulations to poorly soluble APIs as well. In that regard, an incredibly low saturated solubility of IBU in water was determined, i.e., 0.065 mg/mL, corresponding to IBU classification as practically insoluble in water [25]. To overcome solubility limitations, various lipids and surfactants were tested to be used as excipients for SMEDDS, achieving IBU solubility of at least 100 mg/g SMEDDS. Based on the obtained saturated solubility (Table 1), Capmul MCM as the oil phase of SMEDDS with the highest IBU saturated solubility (196.6 mg/g) and Tween 20 or Kolliphor EL as the surfactant phase of SMEDDS with similarly high solubility, i.e., 272.2 and 264.0 mg/g, respectively, were used for further formulation development.

**Figure 1:**
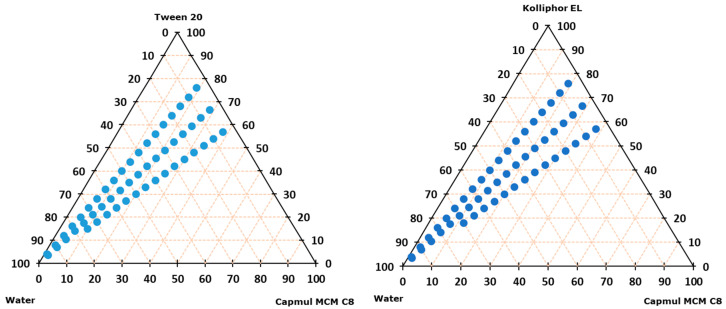
Ternary diagram representing microemulsion formation (blue dot) on water dilution lines of 8:2, 7:3, and 6:4 surfactant–oil mixtures (m/m ratio).

Further, the ability to form microemulsion upon dilution with water was tested for Tween 20/Capmul MCM and Kolliphor EL/Capmul MCM at 8/2, 7/3, and 6/4 weight ratios (Figure 1). For both systems, the microemulsions were formed along the whole dilution line for the 8/2 and 7/3 ratios; however, the 8/2 ratio was chosen for IBU incorporation on the merit of a higher surfactant ratio that additionally reduces the chance of IBU precipitation. At said ratio, the saturated solubility of IBU was 220.2 and 208.9 mg/g in Tween20/Campul MCM and Kolliphor EL/Capmul MCM, respectively.

The possible precipitation of IBU was tested following the incorporation of 150 mg of IBU per g of SMEDDS to avoid the upper solubility limit, and both systems formed microemulsions with no IBU precipitation along the whole dilution line. Nevertheless, Tween 20/Campul MCM was chosen as SMEDDS for further incorporation into hydrogels due to overall higher IBU maximum solubility, with the final compositions of the film-casting dispersions being reported in Table 2.

### 2.2. Rheology of Film Casting Hydrogels

**Figure 2:**
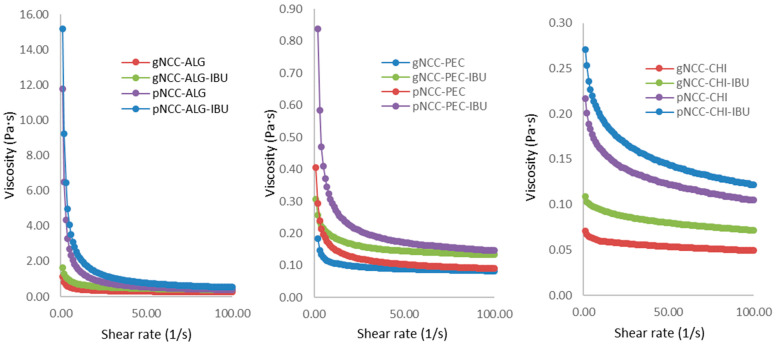
Rotational viscosity of film casting multipolymer hydrogels.

To produce nanocellulose-based films through film casting, gelled dispersions were prepared, cast, and dried. To ensure the films had appropriate properties, such as thickness and film uniformity, these gels were required to exhibit appropriate rheological properties. Our rotational viscosity measurement results (Figure 2) are difficult to directly compare, as the concentration of natural polymers in formulations differed (Table 2). Nevertheless, a clear impact of nanocellulose properties on nanocellulose gels can be seen, as pNCC consistently produced more viscous dispersions compared to gNCC. The reasons for this are shorter (150 nm) and thinner (7.5 nm) polymer fibres attributed to pNCC in comparison to gNCC (150–300 nm length; 15 nm width) coupled with a slightly lesser degree of crystallinity (88% vs. 90.3%, respectively) [17].

The results of the amplitude and frequency sweeps further confirmed rheological differences, with the most prominent impact seen regarding NCC type, i.e., pNCC contributing to more gel-alike behaviour. Namely, gel-like behaviour (G′ > G″) was confirmed in the case of pNCC–ALG, while a liquid-like structure (G″ > G′) could be attributed to gNCC–ALG formulations. Pectin- and chitosan-based hydrogels displayed viscoelastic liquid properties, with weak gel-like behaviour seen only partially for pNCC–PEC hydrogels (Figure S1 in Supplementary Material).

### 2.3. DSC Analysis of IBU-Loaded Films

**Figure 3:**
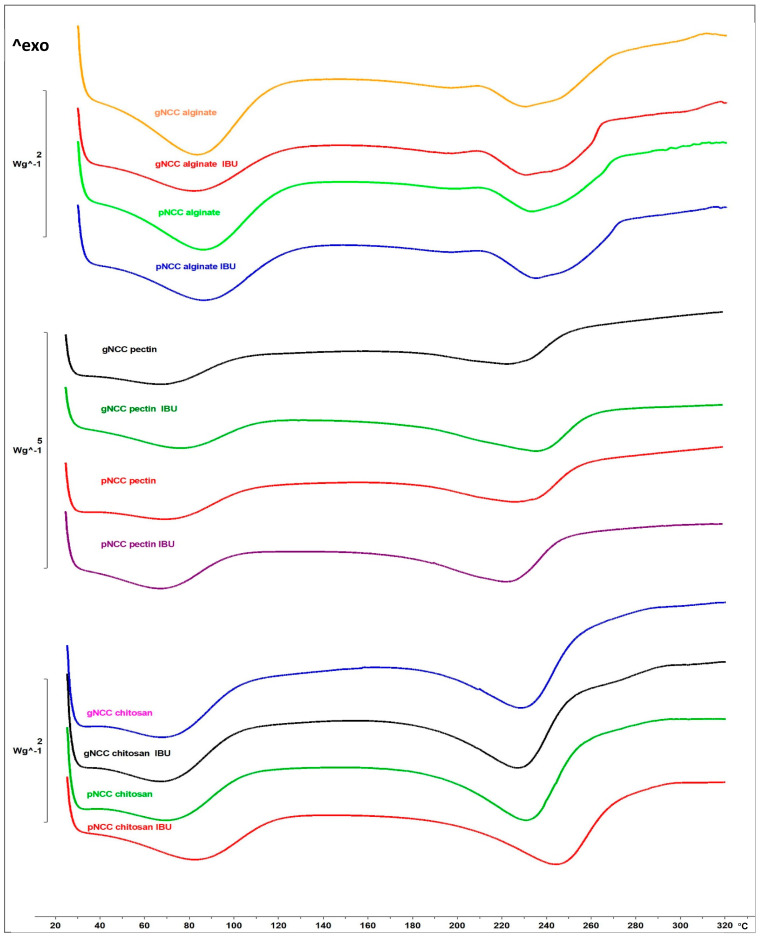
DSC heating curves of unloaded and IBU-loaded buccal films. The y-axis represents a relative heat flux scale, given in W/g of the sample.

DSC analysis of the developed buccal films (unloaded and IBU-loaded) was performed (Figure 3; DSC thermograms of IBU and plain polymers (gNCC, pNCC, alginate, pectin, and chitosan) are presented on Figure S2 in Supplementary Material). No other endothermic peaks, indicative of IBU crystalline form melting, were detected. As the IBU is expected to be dissolved within the film formulation, this enables its rapid absorption following administration to the oral cavity/buccal mucosa. As seen in the ternary diagram (Figure 1), the developed SMEDDS was proven to instantly form a microemulsion any time it came in contact with 5% to 95% (m/m) of added water. Nevertheless, even if there was none or only a minuscule amount of water available in the mouth or within buccal mucosa, the SMEDDS still serves as a solubilising agent to keep IBU in a dissolved state [26]. In this way, when applied to the oral cavity/at the buccal mucosa, IBU can skip the dissolving step and readily absorb through the mucosa. Moreover, surfactants from SMEDDS here act as buccal mucosa penetration enhancers, further impacting the bioavailability of IBU [27].

### 2.4. Thickness and IBU Loading Capacity of Films

While all five studied polymers enabled the successful casting and handling of buccal films, some morphological differences were observed (Figure S3 in Supplementary Material). Namely, gNCC formed more cloudy films in comparison to pNCC, regardless of the type of secondary polymer. Additionally, the surface of the films containing chitosan was rougher in appearance. Due to their more prominent adhesiveness, they were more resistant to removal from the glass surface and harder to cut.

A major obstacle in buccal film development is low API loading capacity due to the limited surface and volume available for such dosage forms. During formulation development, it is important to focus on casting dispersion, enabling the highest content of API, as the final film formulations are limited in both thickness and surface area. Overall, IBU content in films correlated to the viscosity of gels prior to casting, with alginate-based films exhibiting the highest values (>0.8 mg IBU/cm^2^ of film surface) (Table 3). Nevertheless, as the produced films were all under 0.1 mm in thickness, additional improvement in IBU content could be achieved by increasing the viscosity of the gels prior to their casting. On the other hand, thinner films are expected to offer more comfort for patients following their application in comparison to thicker buccal delivery systems [28].

### 2.5. Film Disintegration Behavior

**Figure 4:**
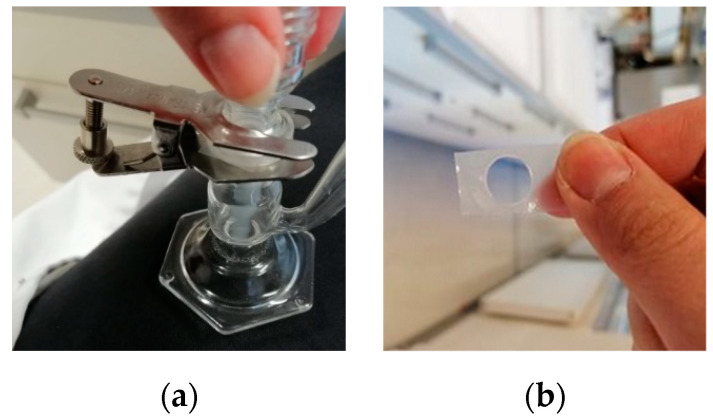
Droplet disintegration study: (**a**) Franz cell set-up; (**b**) example of disintegrated film.

To assess the disintegration time of developed buccal films, two different methods were used: the basket and droplet methods. Overall, the droplet method resulted in longer disintegration times across all evaluated films (Table 4). This was expected, as the volume of water media used is much lower with the droplet method; furthermore, here, only one side of the film is directly exposed to the water vs. the basket method, where the film is thoroughly immersed (Figure 4). Regardless of the method used, however, none of the chitosan-based films managed to disintegrate within 10 min. 

As orally disintegrating films are expected to rapidly disintegrate within 30 s, our disintegration results indicate that, given enough water media, blending nanocellulose with alginate or pectin can produce rapidly disintegrating films. On the other hand, nanocellulose–chitosan-based films are better utilised as buccal films due to their longer perceived disintegration times and the well-regarded mucoadhesion properties of the chitosan. These results were expected, as chitosan is poorly soluble in neutral and basic media [29].

Disintegration results also highlighted the impact of the NCC type incorporated on film properties. Regardless of the natural polymer that was blended with nanocellulose, gNCC consistently produced films that disintegrated more quickly. It can be postulated that gNCC-based films bind water faster and, consequently, disintegrate faster to a lower extent of crosslinking among gNCC and natural polymer chains. As for pNCC-based films, a shorter disintegration time was observed for pNCC–PEC films when compared to pNCC–ALG films, suggesting that the pectin polymer chains intertwine less tightly with pNCC as alginate polymer chains. This is additionally supported by the fastest disintegration time of gNCC–PEC films, even though this also correlates with the lowest thickness of gNCC–PEC films (Table 4). The incorporation of IBU did not significantly impact disintegration time, as the differences perceived were within the standard deviation.

### 2.6. Evaluation of IBU Release from Films

**Figure 5:**
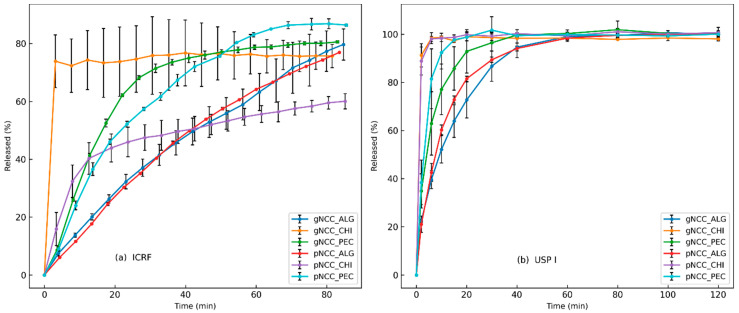
Dissolution testing of NCC formulations: (**a**) ICRF method (left) and (**b**) USP I method.

The results of the dissolution test are shown in Figure 5, where the dissolution rates of the different polymer formulations were compared using two methods. The ICRF method produced more discriminative results compared to USP I, as observed already by Grilc et al. [30]. Chitosan-based films exhibited the fastest dissolution rate, followed by pectin, and Na–alginate-based films showed the slowest release. USP I release rate profiles showed no significant differences between the NCC types used, which was confirmed through bootstrapping evaluation, indicating similarity within the same polymer. The f2 values were 85.7 for chitosan, 54.2 for pectin, and 66.8 for alginate, indicating good similarity between the dissolution profiles within each polymer. However, when the combinations of polymers were evaluated, the f2 values for pectin–alginate (37.0), pectin–chitosan (40.4), and alginate–chitosan (26.7) were less than 50, indicating that the similarity test (f2) was not passed for these combinations. Consequently, it was obvious that the type of polymer had a stronger influence on the dissolution than the type of NCC used. The ICRF method gave comparable results for alginate and pectin formulations, suggesting comparable dissolution behaviours of these two polymers. Surprisingly, the chitosan formulations showed significant differences depending on the type of NCC used. The gNCC type released the drug faster, reaching 75% release after 4 min, while the pNCC type released less than 20% at the same time point. Both chitosan formulations released the drug rapidly initially, in contrast to the pectin and alginate formulations. This observation was unexpected since both NCC–chitosan films did not completely disintegrate during the dissolution test, with incomplete disintegration, as discussed in Section 2.5.

Buccal delivery of ibuprofen was also proposed by Marques et al., who designed a prolonged-release mucoadhesive buccal gel embedded with IBU-loaded lipid nanoparticles. Here, the presented formulation was hydrogel and not film, requiring increased retention time at the mucosa to avoid its clearance by the saliva. In comparison to our buccal film formulations, which followed more closely the conventional release pattern, typical for SMEDDS formulations, the use of a solid nanostructured lipid carrier in the aforementioned hydrogel buccal formulation prolonged the ibuprofen release even further [31].

**Figure 6:**
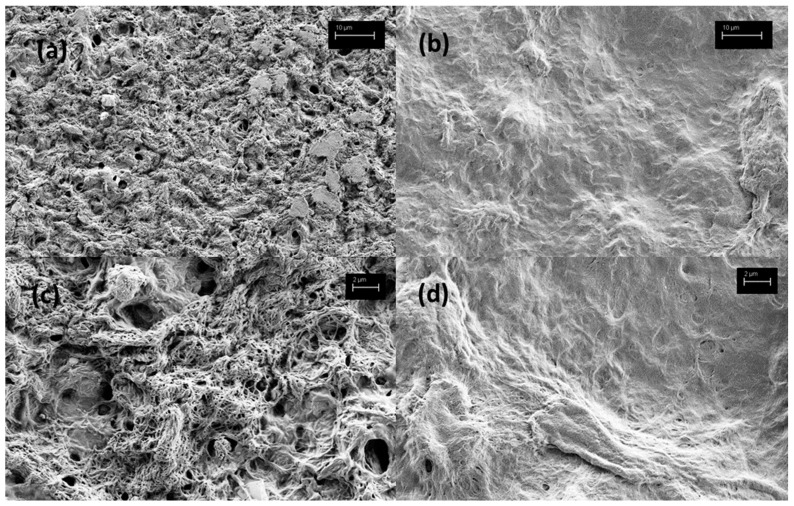
Morphology of remnants of buccal films following dissolution test, under SEM microscopy: (**a**) gNCC–chitosan (3k× magnitude), (**b**) pNCC–chitosan (3k× magnitude), (**c**) gNCC–chitosan (10k× magnitude), (**d**) pNCC–chitosan (10k× magnitude).

Furthermore, the remnants of NCC–chitosan buccal films were further evaluated under SEM following the dissolution of IBU from said films (Figure 6). Due to the high liquid content (SMEDDS and glycerol) of the developed buccal films obscuring resolution, we were unable to observe the morphology of dried buccal films under SEM. This was not the case for remnants of the buccal films following dissolution, however, as they released their liquid components during dissolution testing. Here, a weaker, more porous gel structure is seen on the gNCC–chitosan film compared to pNCC–chitosan (Figure 6a,c vs. Figure 6b,d). This is exacerbated by the dissolution process, as liquid components of films (IBU-loaded SMEDDS, glycerol) are washed out, revealing a looser gel matrix of the gNCC–chitosan vs. pNCC–chitosan films.

## 3. Conclusions

Two types of nanocellulose were used as a novel polymer in combination with natural polymers to successfully develop film for buccal delivery of ibuprofen. Overall, our results highlighted the influence of polymer type on the release profiles, with chitosan showing the fastest dissolution rate, followed by pectin and alginate. Moreover, the choice of NCC significantly influenced the dissolution behaviour of the chitosan formulations. Further development of ibuprofen-loaded nanocellulose buccal films should focus on improving the viscosity of film-casting hydrogels to allow for optimal balance between drug-loading capacity and film thickness. Henceforth, our research work will focus on the investigation of buccal films through in vitro permeation tests on an artificial buccal membrane and buccal cell lines, as well as ex vivo permeation tests across porcine buccal mucosa and in vivo bioavailability following application to the buccal mucosa of New Zealand white rabbits.

## 4. Materials and Methods

### 4.1. Materials

Nanocelluloses used in the study were gNCC (Navitas, Podcerkev, Slovenia) and pNCC (Celluforce, Montreal, QC, Canada). For natural polymers, sodium alginate Protanal^®^ LF 10/60 (ALG) (FMC BioPolymer, Philadelphia, PA, USA), pectin (PEC) (Sigma–Aldrich, St. Louis, MO, USA), and chitosan from low-viscous shrimp shells (CHI) (both from Sigma–Aldrich, St. Louis, MO, USA) were used. IBU was sourced from Fagron (Rotterdam, The Netherlands) and glycerol from Pharmachem Sušnik (Ljubljana, Slovenia). Acetic acid (Merck, Darmstadt, Germany) was added to chitosan-based casting dispersions. Capryol^®^ 90 from Gattefosse (Saint-Priest, France), as well as Kolliphor^®^ EL from BASF (Ludwigshafen, Germany), served as SMEDDS components. Additionally, a solubility study of IBU was performed using the following excipients: oleic acid (Sigma–Aldrich, St. Louis, MO, USA), castor oil (Caesar & Loretz GmbH, Hilden, Germany), Capmul MCM C8 (ABITEC Corporation, Columbus, OH, USA) Tween 20 (Merck, Darmstadt, Germany), Tween 80 (Sigma–Aldrich, St. Louis, MO, USA), Labrasol (Gattefosse, Saint-Priest, France), and Labrafil M2125 CS (Gattefosse, Saint-Priest, France). For High-Performance Liquid Chromatography (HPLC) analysis, all reagents used acetonitrile (Fischer Scientific, Radnor, PA, USA), as well as the sodium hydroxide and sodium dihydrogen phosphate (Merck, Darmstadt, Germany), were analytical grade.

### 4.2. Development of IBU-Loaded SMEDDS

#### 4.2.1. Study of IBU Solubility

To select excipients for the development of IBU-loaded SMEDDS, samples of several lipids (oleic acid, castor oil, Capmul MCM C8) and surfactants (Tween 20, Tween 80, Tween 85, Labrasol, Kolliphor EL, Labrafil M2125 CS) were mixed with a surplus of ibuprofen using a magnetic stirrer for 48 h at 24 ± 1 °C. Afterwards, the samples were centrifuged at 3500 rpm (15 min). An aliquot of supernatant was diluted at a 1:10 (*v*/*v*) ratio with methanol and filtered through a RC 0.45 μm filter before the analysis (Sartorious, Göttingen, Germany), which was used to establish the ibuprofen concentration, with assay performed in duplicates.

#### 4.2.2. HPLC Analysis

An Agilent 1100 Series HPLC system (Agilent Technologies, Santa Clara, CA, USA) equipped with Nucleosil 5 µm C8 100 Å 250 × 4 mm (Phenomenex, Torrance, CA, USA) column was utilised to analyse IBU in 20 μL aliquots of samples. The mobile phase was a mixture of acetonitrile–phosphate buffer (pH 7.4) in a 3:7 ratio (*v*/*v*). The flow rate was adjusted to 1.2 mL/min, and the wavelength of the detector was fixed at 225 nm. Analysis was performed at 25 °C. The calibration curve showed satisfactory linearity in a range from 0.05 to 0.2 mg IBU/mL.

#### 4.2.3. Ternary Phase Diagram Construction

To create a ternary phase diagram, the water titration method, i.e., changing the ratio of oil vs. surfactant (Table 5), was utilised. Oil and surfactant were mixed in a specific ratio to obtain a transparent and homogenous mixture. Afterwards, water in increments of 5% (m/m) was added to a total of 95% (m/m). The resulting dispersions were visually observed for turbidity, by which the boundaries of the microemulsion domain were determined.

#### 4.2.4. Microemulsion Loading with Ibuprofen

A total of 150 mg of IBU was dissolved in 1 g of SMEDDS, a combination of oil and surfactant from Section 4.2.3 with a confirmed ability to form microemulsion upon the addition of water in the entire range from 5% to 95% (m/m). The resulting IBU-loaded SMEDDS was diluted, with water representing 95% (m/m) of the final dispersion as well. Afterwards, said dispersion was centrifuged (10 min at 3500 rpm) to exclude the possibility of phase separation or IBU precipitation.

### 4.3. Development of Buccal Film Formulations

Firstly, to prepare nanocellulose-based film casting dispersions with added alginate or pectin, Tween 20 and Capmul MCM (at 8:2 m/m ratio) were blended with IBU to prepare IBU-loaded SMEDDS. Subsequently, water was added to form a microemulsion. To the resulting microemulsion, NCC was added while magnetically stirring until the formation of the homogenous mixture. Thereafter, alginate or pectin powder was sprinkled, and the dispersions were left to mix in a covered beaker for 12 h. Finally, glycerol was added, and a final mixing of 30 min was performed.

For the nanocellulose–chitosan-based dispersions, the procedure was slightly adapted due to the insolubility of chitosan in neutral media; here, microemulsions were prepared from SMEDDS with the addition of 1% acetic acid instead of pure water. The rest of the procedure mirrored the preparation of dispersions described above, the only difference being the addition of pectin in place of alginate or pectin. Moreover, the preparation of film-casting dispersions without the API was conducted using the same technique, except the incorporation of IBU into the microemulsions was omitted.

Buccal films were prepared using the solvent casting method. Briefly, film casting dispersions were evenly distributed across glass plates by means of a universal film applicator (ZUA 2000; Proceq, Schwerzenbach, Switzerland). The dispersion layer thickness was adjusted to 2 mm. Subsequently, dispersions were dried (SP-45 dryer, Kambič, Semič, Slovenia) for 4 h at 60 °C and left to cool at room temperature before cutting into 3 cm × 1.5 cm strips. Until further use, films were individually stored in heat-sealed aluminium foil pouches at room temperature (22 ± 2 °C).

#### 4.3.1. Rheological Study

The rotational method was used to determine the rheological properties of the developed hydrogels prior to film casting and was carried out at 25 °C in a Physica MCR 301 rheometer (Anton Paar, Graz, Austria) equipped with a conical disk attachment (49.961 mm radius; cone angle of 2.001°). Approximately 1 g of the sample was applied and left to rest for 30 s. Afterwards, the shear rate was amplified from 1 to 100 (1/s); the shear stress measurements were evaluated with Physica software.

For the amplitude test, the angular velocity was kept at 10 rad/s while the shear strain was continuously amplified from 0.01 to 100% and while the elastic (G′) and plastic (G″) moduli were recorded.

To perform frequency tests, a previously determined range of linear viscoelastic responses was utilised. Therefore, changes in both the elastic (G′) and plastic (G″) moduli at 0.1% deformation and angular velocity of 100–0.1 rad/s were monitored.

#### 4.3.2. DSC Analysis

DSC method (DSC1, Mettler Toledo, Columbus, OH, USA) was utilised for the physical state characterisation of IBU in developed buccal films. Briefly, approximately 2 mg of buccal film samples (with or without IBU) were accurately weighed and placed into an aluminium pan. Prior to the analysis, the pan was covered with a lid, hermetically sealed, and pierced. Scanning of the samples was performed within the 25 °C to 320 °C temperature rate and under a nitrogen flow of 50 mL/min with the heating rate set at 10 °C/min. STARe V9.30 software (Mettler Toledo, Columbus, OH, USA) was used to normalise DSC curves to the weight of samples and determine the melting points (Tm, peak).

#### 4.3.3. Film Thickness Measurement

After drying, a sharp blade was utilised to cut films of approximately 3 cm × 1.5 cm in size. A digital calliper was used to measure the thickness of each film sample in three separate places.

#### 4.3.4. Total Content of IBU in Films

To determine the total IBU content, the films were left to completely dissolve in 1 mL of bidistilled water with constant stirring for 20 min. Prior to HPLC analysis, the samples were diluted with methanol, filtered through a Sartorius RC 0.2 µm membrane filter, and analysed in four parallels.

### 4.4. Determination of Disintegration Time

#### 4.4.1. Basket Method

The disintegration time of buccal films was determined using the EP/USP/JP-compliant disintegration tester Erweka ZT4 (Erweka GmbH, Langen, Germany). Next, 700 mL of purified water heated to 37 °C ± 2 °C served as the media. A 1.5 cm × 1.5 cm piece of film was placed within each test tube of Basket type A. Disintegration time of developed film formulations with and without IBU was determined within sextuplicate measurements.

#### 4.4.2. The Droplet Method

The disintegration time of prepared buccal films was additionally determined using the drop method. A buccal film cut to 1.5 cm × 1.5 cm dimension was mounted between the donor and acceptor chamber of a vertical Franz cell. Subsequently, 200 μL of purified water was applied to the film. The time for the droplet to flow through film samples was measured in quadruplicates. In the case where the recorded time exceeded 10 min, an additional aliquot of 200 μL purified water was added to the film.

### 4.5. IBU Release from Films

#### 4.5.1. USP I Method

Film dissolution tests were conducted using a USP I basket method apparatus (Varian VK 7000, Agilent Technologies, Santa Clara, USA) with a 50 rpm rotational speed of 50 rpm to facilitate drug release. A 0.1 M sodium phosphate buffer with a pH of 6.8 served as the dissolution medium, with a total volume of 500 mL. Samples were collected at predetermined intervals: 2, 6, 10, 15, 20, 30, 40, 60, 80, 100, and 120 min. For each sampling, 6 mL of the medium was collected and subsequently filtered through RC membrane filters of 0.45 µm pore size to be analysed using HPLC. Next, 6 mL of fresh buffer solution was subsequently added to the dissolution media following each sample withdrawal. The films were vertically positioned in the basket and mounted on the rotating rods immediately before the dissolution test commenced to prevent the absorption of moisture, which could impact dissolution performance.

#### 4.5.2. Flow-Through Dissolution Method

In 2020, our research team introduced an innovative cell for film release evaluation, the “Innovative Cell for Film Release” (ICFR), which was described in detail in the publication [32]. This system consists of a two-chamber flow cell separated by a membrane with two inlets and one outlet. To hold the film securely during evaluation and maintain continuous contact with the membrane, the film is positioned on the surface of the membrane, and the edges of the cell chamber provide its fixation. A cellulose acetate membrane with 0.45 µm pores (Sartorius, Göttingen, Germany) was utilised to support the film. A hydrostatic pressure of 490 Pa or higher was sustained within the donor chamber vs. the acceptor chamber. This configuration, combined with laminar fluid flow, naturally extends dissolution times compared to other methods, making it advantageous for detecting subtle differences between formulations. The medium flow rate through the acceptor chamber was maintained at 20 mL/min. The inlet medium was preheated, and the temperature within the flow cell was kept at 37 °C. Precise temperature was recorded at intervals of 1 s. To ensure consistent and accurate sample collection, we used an autosampler to collect samples at four-minute intervals. A 0.1 M sodium phosphate buffer of pH 6.8 was used as a medium here as well.

#### 4.5.3. Dissolution Results Analysis

A model-independent approach, adhering to international guidelines, was used to evaluate dissolution by calculating the f2. Through bootstrapping, a 90% confidence interval for similarity factor f2 was determined (n = 5000). To deem the profiles similar, the lower bound of the confidence interval for f2 had to exceed 50. All calculations were conducted using the Excel add-in DDSolver 1.0. [33].

### 4.6. Scanning Electron Microscopy

Morphological differences of NCC–chitosan buccal films placed on a carbon tape were observed under a Supra 35 VP scanning electron microscope (SEM) at high resolution (Carl Zeiss, Oberkochen, Germany) at different magnifications using a secondary detector and 1.0 kV of acceleration voltage.

## Figures and Tables

**Table 1:** Saturated solubility of IBU in selected excipients.

Phase Type	Excipient	Saturated Solubility of IBU [mg/g]
Surfactant	Tween 20	272.16
	Kolliphor EL	264.00
	Labrasol	258.50
	Tween 80	254.66
	Tween 85	225.40
	Labrafil M2125 CS	123.14
Oil	Capmul MCM	196.58
	Oleic acid	150.27
	Castor oil	139.64

**Table 2:** Compositions of film-casting hydrogels in m/m % (purified water was added to 100% ^1^).

SAMPLE	gNCC/pNCC	ALG/PEC/CHI	Glycerol	Ibuprofen	SMEDDS
gNCC–ALG	1%	2%	3%	/	3.4%
gNCC–ALG–IBU	1%	2%	3%	0.6%	3.4%
pNCC–ALG	1%	2%	3%	/	3.4%
pNCC–ALG–IBU	1%	2%	3%	0.6%	3.4%
gNCC–PEC	0.6%	1.2%	3%	/	3.4%
gNCC–PEC–IBU	0.6%	1.2%	3%	0.6%	3.4%
pNCC–PEC	0.57%	1.15%	3%	/	3.4%
pNCC–PEC–IBU	0.57%	1.15%	3%	0.6%	3.4%
gNCC–CHI	0.5%	1%	3%	/	3.4%
gNCC–CHI–IBU	0.5%	1%	3%	0.6%	3.4%
pNCC–CHI	0.5%	1%	3%	/	3.4%
pNCC–CHI–IBU	0.5%	1%	3%	0.6%	3.4%

^1^ 1% acetic acid was added to chitosan-based films.

**Table 3:** Total content of IBU in films cut to 3 cm × 1.5 cm size.

Sample	Thickness [mm]	Total Weight [mg]	IBU Content [mg/per Film]	IBU/Film [%, m/m]	mg IBU/cm^2^ Film Surface
gNCC–ALG–IBU	0.10 ± 0.01	62.54 ± 3.61	3.71 ± 0.14	5.93	0.82
pNCC–ALG–IBU	0.10 ± 0.01	70.92 ± 3.68	3.60 ± 0.37	5.07	0.80
gNCC–PEC–IBU	0.05 ± 0.01	28.92 ± 1.29	1.76 ± 0.07	6.08	0.39
pNCC–PEC–IBU	0.07 ± 0.01	55.74 ± 4.08	2.43 ± 0.26	4.35	0.54
gNCC–CHI–IBU	0.02 ± 0.01	9.72 ± 1.09	1.86 ± 0.27	19.14	0.41
pNCC–CHI–IBU	0.03 ± 0.01	20.93 ± 1.64	0.91 ± 0.14	4.34	0.20

**Table 4:** Disintegration time of films as observed through the basket or droplet method.

Sample	Disintegration Time Basket Method [s]	Disintegration Time Droplet Method [s]
gNCC–ALG	22.0 ± 3.5	112.8 ± 18.6
gNCC–ALG–IBU	26.0 ± 2.1	120.5 ± 6.0
pNCC–ALG	57.3 ± 5.6	451.0 ± 78.5
pNCC–ALG–IBU	56.0 ± 5.0	462.8 ± 74.6
gNCC–PEC	18.5 ± 1.0	31.5 ± 2.6
gNCC–PEC–IBU	17.3 ± 2.2	95.5 ± 12.7
pNCC–PEC	29.8 ± 2.1	220.5 ± 34.7
pNCC–PEC–IBU	29.5 ± 1.3	221.3 ± 25.5

**Table 5:** Composition of ternary phase mixtures.

Lipid Phase	Surfactant	Lipid/Surfactant Ratio
Capmul MCM	Tween 20	2/8
		3/7
		4/6
	Kolliphor EL	2/8
		3/7
		4/6

## Data Availability

The original contributions presented in the study are included in the article/Supplementary Materials. Further inquiries can be directed to the corresponding author.

## Supplementary Materials

The following supporting information can be downloaded at: https://www.mdpi.com/article/10.3390/gels11030163/s1, Figure S1: Amplitude (a), (c), (e) and frequency (b), (d), (f) sweeps of multipolymer casting hydrogels; Figure S2: DSC heat curves of ibuprofen and plain polymers (gNCC, pNCC, alginate, pectin, and chitosan). The y-axis represents a relative heatflux scale given in W/g of the sample; Figure S3: Macroscopic appearance of developed multipolymer buccal films.
