# Supplementary material for: Ibuprofen-Loaded, Nanocellulose-Based Buccal Films: The Development and Evaluation of Promising Drug Delivery Systems for Special Populations
Source: Gels. 2025 Feb 24;11(3):163. doi: 10.3390/gels11030163 (PMC11942018; doi:10.3390/gels11030163)
Supplement: Supplementary file 1 [file gels-11-00163-s001.zip › gels-3373723-supplementary.pdf]

SUPPLEMENTARY MATERIAL for the study

Ibuprofen loaded nanocellulose-based buccal films: development and evaluation of promising drug delivery systems for special populations

Katarina Bolko Seljak[1], Blaž Grilc[1,] Mirjana Gašperlin[1] and Mirjam Gosenca Matjaž[1]

[1] University of Ljubljana, Faculty of Pharmacy, Aškerčeva cesta 7, 1000 Ljubljana, Slovenia

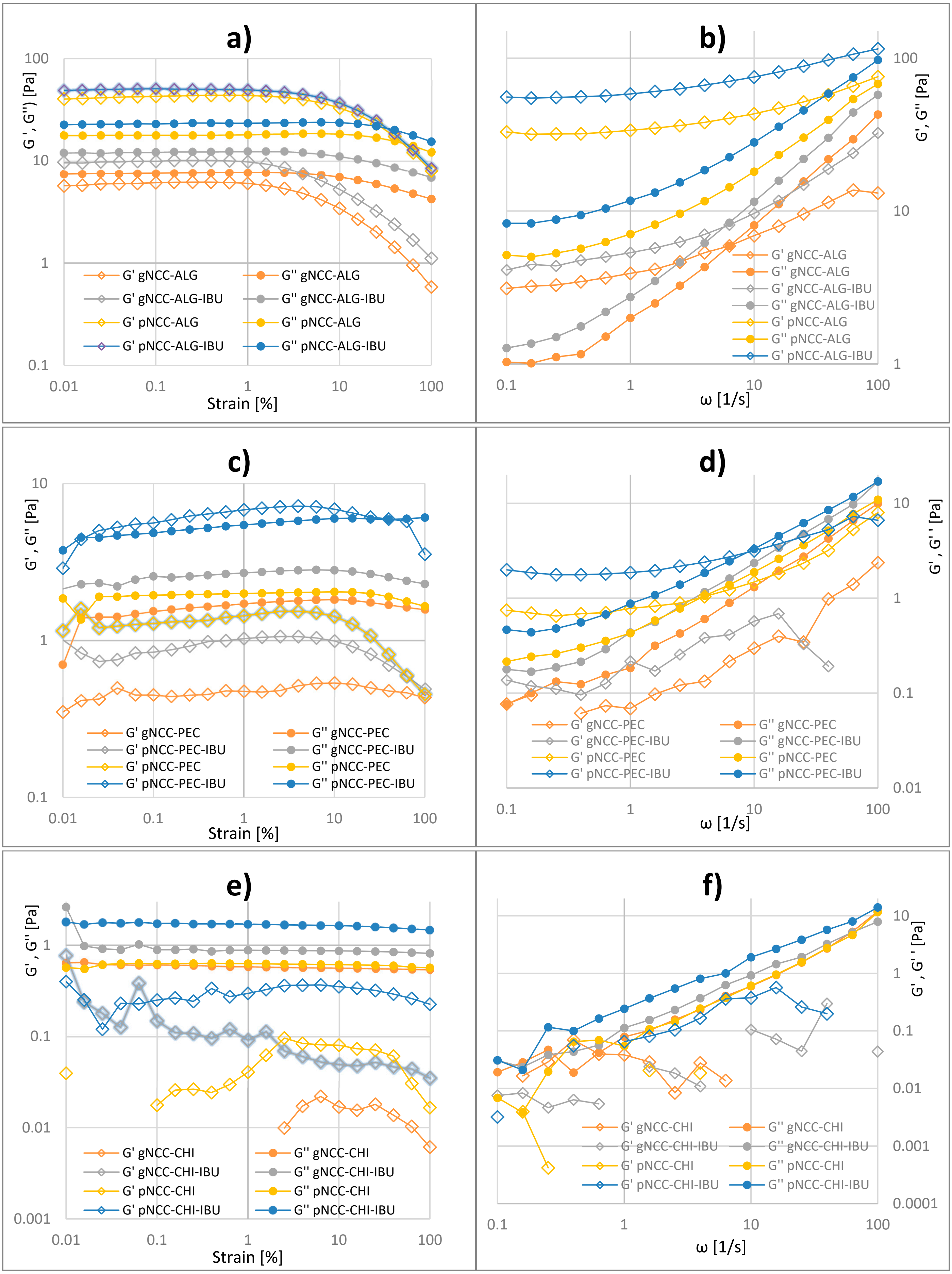

Figure S1: Amplitude (a), c), e)) and frequency (b), d), f)) sweeps of multipolymer casting hydrogels.

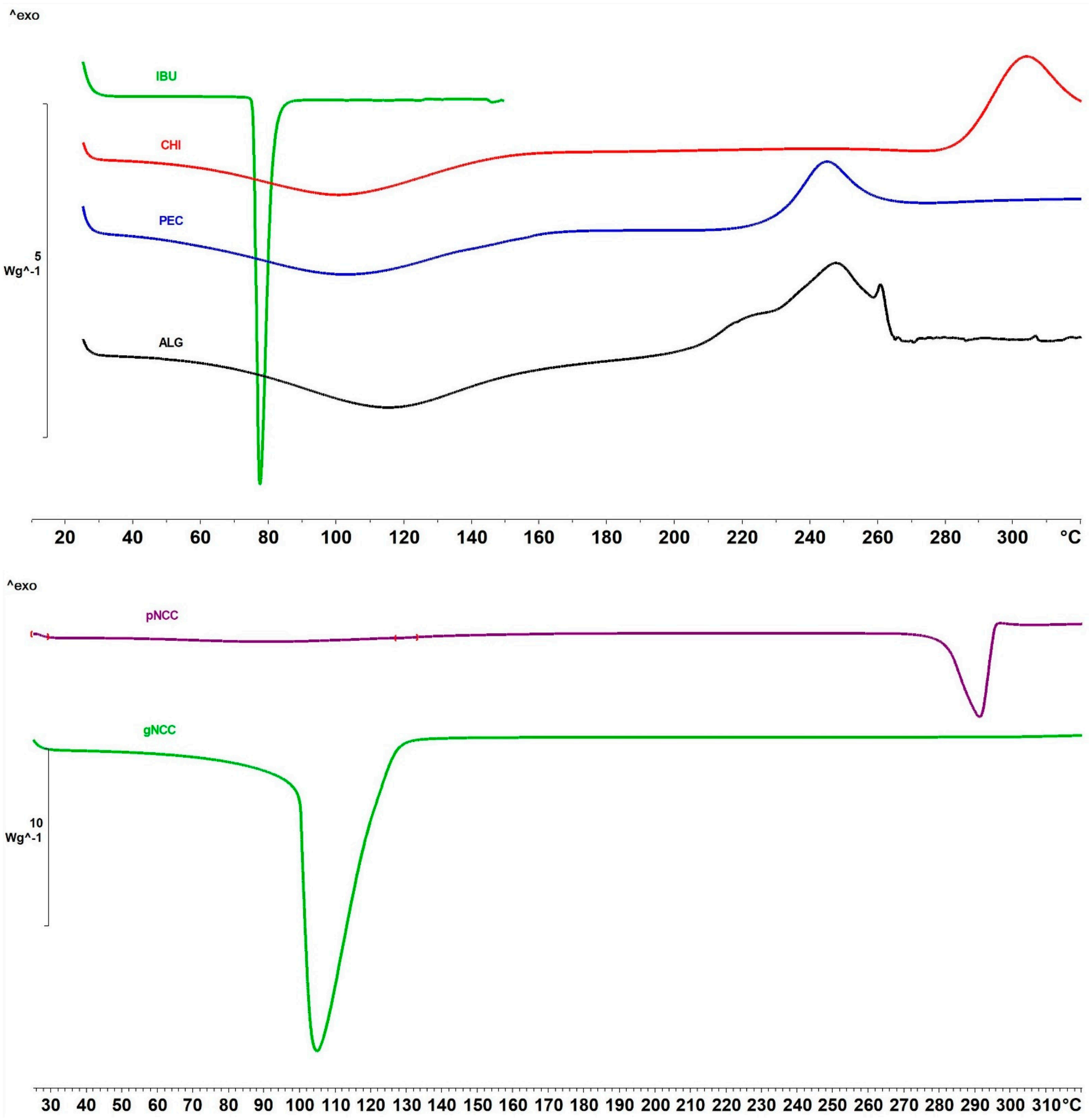

Figure S2: DSC heat curves of ibuprofen and plain polymers (gNCC, pNCC, alginate, pectin, and chitosan). The y-axis represents a relative heatflux scale given in W/g of the sample.

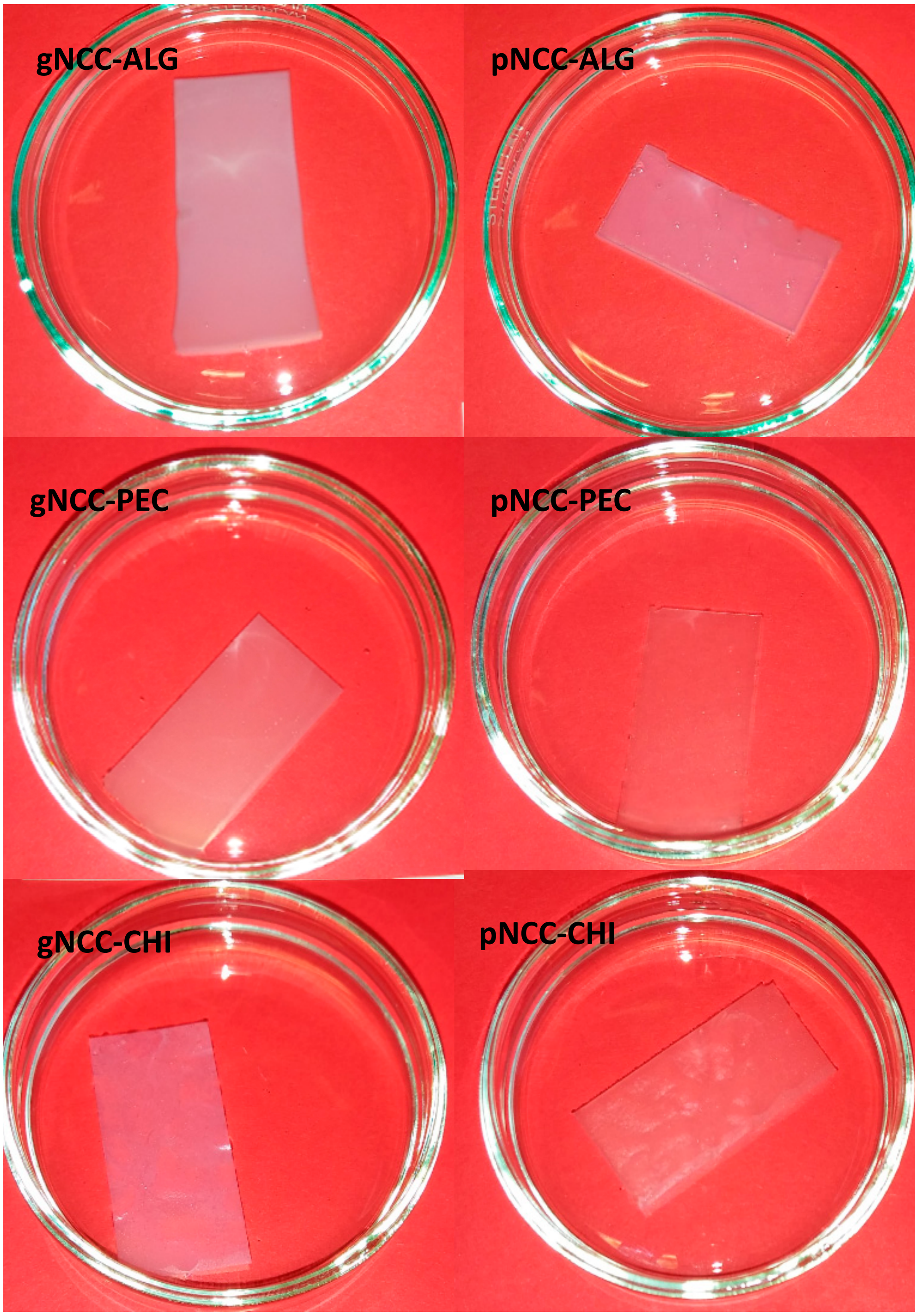

Figure S3: Macroscopic appearance of developed multipolymer buccal films.
